# Virus-Induced Gene Silencing Identifies an Important Role of the *TaRSR1* Transcription Factor in Starch Synthesis in Bread Wheat

**DOI:** 10.3390/ijms17101557

**Published:** 2016-09-23

**Authors:** Guoyu Liu, Yufang Wu, Mengjun Xu, Tian Gao, Pengfei Wang, Lina Wang, Tiancai Guo, Guozhang Kang

**Affiliations:** 1 The Collaborative Innovation Center of Henan Food Crops, Henan Agricultural University, Zhengzhou 450002, China; liuguoyu@henau.edu.cn (G.L.); yufangwu628@henau.edu.cn (Y.W.); wangpf@henau.edu.cn (P.W.); hnndlina@henau.edu.cn (L.W.); 2 The National Key Laboratory of Wheat and Maize Crop Science, Henan Agricultural University, Zhengzhou 450002, China; mengjunxu920304@henau.edu.cn (M.X.); gaotian1211@henau.edu.cn (T.G.); 3 The National Engineering Research Centre for Wheat, Henan Agricultural University, Zhengzhou 450002, China

**Keywords:** *TaRSR1*, *Triticum aestivum* L., BSMV-VIGS, starch synthesis regulation

## Abstract

The function of a wheat starch regulator 1 (*TaRSR1*) in regulating the synthesis of grain storage starch was determined using the barley stripe mosaic virus—virus induced gene-silencing (BSMV-VIGS) method in field experiments. Chlorotic stripes appeared on the wheat spikes infected with barley stripe mosaic virus-virus induced gene-silencing- wheat starch regulator 1 (BSMV-VIGS-TaRSR1) at 15 days after anthesis, at which time the transcription levels of the *TaRSR1* gene significantly decreased. Quantitative real-time PCR was also used to measure the transcription levels of 26 starch synthesis-related enzyme genes in the grains of BSMV-VIGS-TaRSR1-silenced wheat plants at 20, 27, and 31 days after anthesis. The results showed that the transcription levels of some starch synthesis-related enzyme genes were markedly induced at different sampling time points: *TaSSI*, *TaSSIV*, *TaBEIII*, *TaISA1*, *TaISA3*, *TaPHOL*, and *TaDPE1* genes were induced at each of the three sampling time points and *TaAGPS1-b*, *TaAGPL1*, *TaAGPL2*, *TaSSIIb*, *TaSSIIc*, *TaSSIIIb*, *TaBEI*, *TaBEIIa*, *TaBEIIb*, *TaISA2*, *TaPHOH*, and *TaDPE2* genes were induced at one sampling time point. Moreover, both the grain starch contents, one thousand kernel weights, grain length and width of BSMV-VIGS-TaRSR1-infected wheat plants significantly increased. These results suggest that TaRSR1 acts as a negative regulator and plays an important role in starch synthesis in wheat grains by temporally regulating the expression of specific starch synthesis-related enzyme genes.

## 1. Introduction

Starch is the major storage carbohydrate reserve in the endosperm of cereals, including rice, wheat, and maize, and provides about 80% of the calories consumed by humans [1]. Plant starch is composed of two different glucose polymers: amylose and amylopectin. The former is a linear polymer composed of α-1,4-glucosidic chains, whereas the latter consists of a highly branched glucan with α-1,6 glucosidic bonds that connect linear chains [2]. Amylose is synthesized by adenosine diphosphate glucose pyrophosphorylase (AGPase, EC 2.7.7.27) and granule-bound starch synthase (GBSS, EC 2.4.1.21), whereas amylopectin is catalyzed by the coordinated actions of AGPase, soluble starch synthase (SS, EC 2.4.1.21), starch branching enzyme (BE, EC 2.4.1.18), starch debranching enzyme (DBE) (isoamylase, ISA, EC 3.2.1.68; pullulanase, PUL, EC 3.2.1.41), disproportionating enzyme (DPE, EC 2.4.1.25), and phosphorylase (PHO, EC 2.4.1.1) [2]. AGPase catalyzes adenosine diphosphate glucose (ADP-Glc) to glucose-1-phosphate (G-1-P) and provides substrates for amylose and amylopectin synthesis. GBSS is a key enzyme involved in amylose synthesis, whereas SS, BE, and DBE function together with distinct roles to catalyze amylopectin synthesis [2,3]. In starch biosynthesis, PHO and DPE are believed to be involved in the initiation steps, elongating α-1,4-linked glucan polymers using G-1-P as substrate, although their precise mechanisms remain unclear [2,4].

Most starch synthesis-related enzymes have multiple subunits or isoforms in higher plants, and the number of subunits or isoforms for each enzyme is plant species-specific [5]. In the rice genome, 11 and 3 isozymes of SS and BE, respectively, have been identified [5]; in the maize genome, 9 and 4 isozymes of SS and BE, respectively, have been identified [6]; and in wheat, rice, and maize, 26, 29, and 32 starch synthesis-related enzyme genes, respectively, have been identified [5,6,7]. Temporal and spatial coordination of these starch synthesis-related enzymes may be important for converting photosynthetic products to starch and grain storage organs. To some extent, these genes are differentially expressed among plant species because there are some differences in the functional properties of starch [8]. Comprehensive transcription profiles of starch synthesis-related enzyme genes in rice, barley, maize and wheat have been determined, and of them, key genes that play essential roles in endosperm starch synthesis have been identified [5,6,7,9].

Previous studies have focused on identifying the expression profiles and effects of single starch synthesis-related enzyme genes in starch biosynthesis [2]; however, the molecular machinery regulating the expression of these genes remains unclear despite the fact that this is a finely regulated process. Transcription factors play important roles in plant growth, development, and abiotic and biotic stress responses because they specifically bind to *cis*-acting elements in the promoter region of target genes [10]. Most of the transcription factors isolated from higher plants are involved in abiotic and biotic stress responses, whereas few have been associated with starch synthesis [11,12,13].

Bread wheat (*Triticum aestivum* L.), one of the major staple crops for the human diet, is an essential component of the global food security [14]. It has a large, complex, and allohexaploid genome consisting of A, B, and D subgenomes (2*n* = 6*x* = 42, AABBDD; 2*n* is the number of chromosomes in each somatic cell and 6× is the basic chromosome); therefore, each wheat gene potentially exists as a trio of A, B, and D homoeoloci [15]. However, there are complicated regulatory mechanisms within the cells of an allopolyploid genome, and through genetic or epigenetic modifications, these mechanisms can orchestrate the complex intergenomic gene expression [15,16]. The genetic or epigenetic modifications have brought genomic asymmetry toward a diploid-like mode of expression, because either of mutation, elimination or repression of most genes that, in most cases, confine the activity of sets of genes to only one genome [17]. With the recent release and annotation of the bread wheat genome, it is now possible to expand better characterize its growth, development, and responses to biotic and abiotic stresses [18,19].

The euAPETALA2 (euAP2) group of the APETALA2/ethylene-responsive element binding protein (AP2/EREBP) family is characterized by the APETALA2 (AP2) domain, and some members of this group function in the boundaries of floral organ identity, floral meristem identity, or the control of floral organ number [20,21,22,23]. In our previous study, transcription levels of *Rice* *Starch Regulator 1* (*RSR1*), an euAP2 group transcription factor, was significantly and negatively associated with the transcription levels of 11 starch synthesis-related enzyme genes during the wheat grain-filling period, suggesting that *TaRSR1* may negatively regulate the expression of some starch synthesis-related enzyme genes in wheat grains [7]. In bread wheat, particle bombardment and co-cultivation with *Agrobacterium tumefaciens* have been used to explore the function of candidate genes. However, the disadvantages of these two methods include multiple copy insertions, cultivar-specificity, time consumption, and low transformation efficiency; this species may be the last cereal to be genetically transformed [23,24]. The barley stripe mosaic virus–virus induced gene silencing (BSMV-VIGS) was recently developed as an attractive tool for the rapid generation of gene knockdown phenotypes in plants. The technique mainly exploits the BSMV-mediated antiviral defense mechanism of plants to study the function of endogenous genes based on homology-dependent gene silencing. It has played an important role in the functional identification of genes in bread wheat because it is not required for plant transformation and can potentially accelerate the characterization of target genes [25,26]. In this study, BSMV-VIGS method was used to further verify the above-mentioned hypothesis. Our results indicate that *TaRSR1* may negatively and temporally regulate the expression of some starch synthesis-related enzyme genes, thereby supporting its regulatory role in wheat starch synthesis.

## 2. Results

### 2.1. Phenotypes of Barley Stripe Mosaic Virus-Wheat Starch Regulator 1 (BSMV-TaRSR1) Infected Wheat Plants

Our previous study indicated that, in bread wheat, starch synthesis-related enzymes are encoded by 26 genes: AGPase (*TaAGPS1-a*, *TaAGPS1-b*, *TaAGPS2*, *TaAGPL1*, and *TaAGPL2*), GBSS (*TaGBSSI* and *TaGBSSII*), SS (*TaSSI*, *TaSSIIa*, *TaSSIIb*, *TaSSIIc*, *TaSSIIIa*, *TaSSIIIb* and *TaSSIV*), *BE* (*TaBEI*, *TaBEIIa*, *TaBEIIb* and *TaBEIII*), DBE (*TaISA1*, *TaISA2*, *TaISA3* and *TaPUL*), PHO (*TaPHOL* and *TaPHOH*), and DPE (*TaDPE1* and *TaDPE2*). Fu and Xue [22] reported that the transcription levels of *OsRSR1* were almost opposite those of 15 rice starch synthesis-related enzyme genes (*OsAGPS1*, *OsAGPS2*, *OsAGPL1*, *OsAGPL2*, *OsGBSSI*, *OsSSI*, *OsSSIIa*, *OsSSIIIa*, *OsBEI*, *OsBEIIb*, *OsISA1*, *OsISA2*, *OsISA3*, *OsPHOL*, and *OsPUL*), and in an *rsr1* mutant, these genes were distinctly upregulated during the early stage (6 days) of the grain-filling period when grain starch contents significantly increased. Our previous data indicated that the transcription levels of TaRSR1 were significantly negatively correlated with those of 11 starch synthesis-related enzyme genes (*TaAGPS1-a*, *TaAGPL1*, *TaGBSSI*, *TaSSI*, *TaSSIIa*, *TaSSIV*, *TaBEI*, *TaBEIIb*, *TaPUL*, *TaPHOL*, and *TaDPE1*) during the entire grain-filling period (35 days) of bread wheat, suggesting that (similar to *OsRSR1*) *TaRSR1* negatively regulates the expression of these genes and affects grain starch synthesis. However, there may be some differences (e.g., target genes, regulatory stages) in the regulatory mechanisms of RSR1 involved in starch synthesis between bread wheat and rice [7]. In this study, the function of TaRSR1 in the starch synthesis of wheat grains was further explored.

Higher plants including important crops are grown under field conditions with natural variable factors (e.g., temperature, light illumination and humidity). Thus, field experiments with variable growth factors may more accurately measure the function of candidate genes than controlled environments with designed growth factors [27]. The BSMV-VIGS method was previously used to explore the function of candidate genes of cereal crops under controlled environments [13,28,29]. Here, this method was used in field experiments to explore the function of TaRSR1. Humidity is a major growth factor that affects the efficiency of the BSMV-VIGS method [30]. To increase humidity, wheat plants grown under field conditions were covered with an arch plastic shed at the heading stage, and wheat plants in the shed were sprayed with distilled water at 1 d before inoculation. Thus, relative humidity in the arch plastic shed was close to 85.0%, which was suitable for BSMV-VIGS inoculation [30].

**Figure 1:**
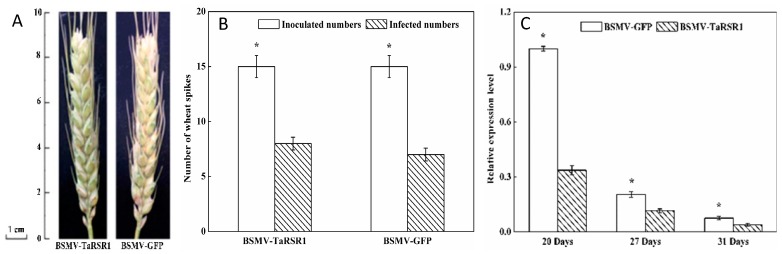
Phenotypes (**A**); number (**B**); and *TaRSR1* gene transcription levels (**C**) of the BSMV-TaRSR1-infected and BSMV-GFP-infected wheat spikes. Appearance of chlorosis on the inoculated wheat spikes at 22 days after anthesis suggests that BSMV virus has been infected and the target genes are successfully silenced by VIGS in this tissue. Efficiency of the BSMV-VIGS method was based on chlorosis phenotype, and the average values of the number of VIGS infection from 15 plants were considered as one replicate and three independent biological replicates were performed. Transcription levels at 20, 27 and 31 days after anthesis were measured by qPCR using *Actin* gene as internal control. Each value is the mean ± standard deviation of three independent biological replicates. Asterisks indicate significant differences (*p* < 0.05).

When a fragment of a plant gene is inserted into virus induced gene-silencing (VIGS) vector, a recombinant virus is formed, and after a plant host is infected, this recombinant virus can induce post-transcriptional gene silencing targeting both the virus RNA and homologous endogenous plant RNA sequences for degradation. Only plant barley stripe mosaic virus (BSMV), a tri-partite RNA genome comprising RNAα, RNAβ and RNAγ, has so far been developed into the VIGS vector, and there appears chlorosis induced by BSMV virus on the inoculated tissues [31,32]. In VIGS experimental design, at least one negative control VIGS construct containing GFP or β-glucuronidase gene (GUS) should be included, and appearance of chlorosis on the inoculated tissues suggests that BSMV virus is inoculated and the target genes are silenced by VIGS in plant host [33]. In this study, a fragment (284 bp) of the *TaRSR1* gene (GenBank accession no. JX473823) was isolated (Figure S1), the BSMV-derived *TaRSR1* silencing vector was constructed, which was used to inoculate the spikes of 45 plants grown in the arch plastic shed during the heading stage under field conditions. BSMV-derived green fluorescent protein (GFP)-silenced wheat plants were used as the control. Our data indicated that BSMV-VIGS-induced chlorosis appeared in the middle of the wheat spikes at 15 days after anthesis, after which it extended throughout the entire spikes and persisted until the mature grain stage (Figure 1A). This suggested that TaRSR1 was successfully silenced in these wheat spikes. Of the 45 BSMV-TaRSR1-infected or BSMV-GFP-infected wheat spikes, 25 and 22 showed chlorosis with silencing efficiencies of 55.6% and 48.9% (average of 52.5%), respectively (Figure 1B).

### 2.2. Transcription Levels of TaRSR1 in BSMV-TaRSR1 Infected Wheat Plants

These qualitative phenotypic results were further confirmed by quantitative analysis of the expression levels of the *TaRSR1* gene using quantitative real-time PCR (qPCR) at 20, 27, and 31 days after anthesis. Wheat *β-actin* gene was used as an internal control. Similar data were obtained using the glyceraldehyde 3-phosphate dehydrogenase (*GAPDH*) gene as another internal control, which is shown in Figure S2. Our results showed that transcription levels of the *TaRSR1* gene in the grains of BSMV-TaRSR1 infected spikes were markedly decreased by 43.5%–66.3% at the three sampling time points (Figure 1C). The degree of suppression was in accordance with previous data in wheat [34], thereby supporting the fact that the *TaRSR1* gene is silenced in the grains of wheat plants grown under field conditions. In this study, the efficiency (52.5%) of the BSMV-VIGS method used in wheat plants under field conditions was far lower than that (90%) in experiments performed by Ma et al. [28] under greenhouse conditions. This lower efficiency may be due to the variable growth parameters under field conditions including humidity, light illumination, and temperature during the reproductive period of BSMV virus inoculation [30,35].

### 2.3. Starch Contents and One Thousand Kernel (1000-Kernel) Weights in BSMV-TaRSR1 Infected Wheat Plants

**Figure 2:**
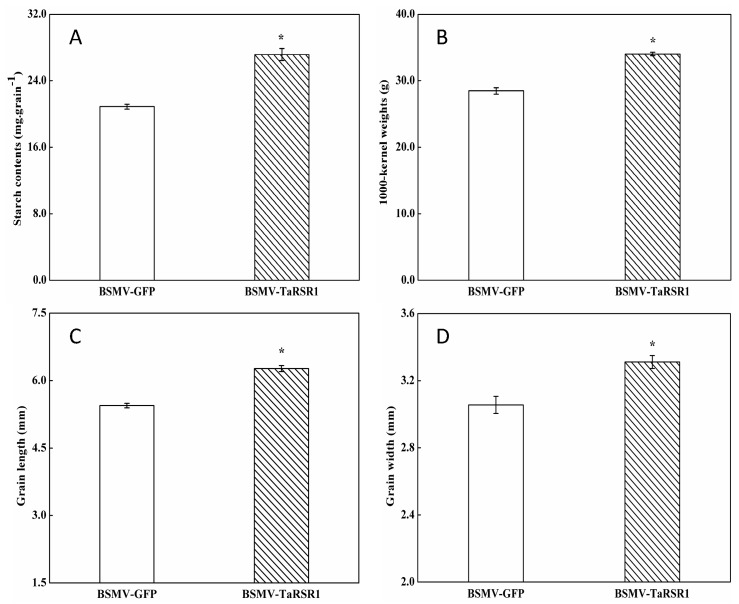
Matured grain starch contents (**A**); 1000-kernel weights (**B**); length (**C**); and width (**D**) of BSMV-TaRSR1-infected and BSMV-GFP-infected wheat spikes. Each value is the mean ± standard deviation of three independent biological replicates. Asterisks indicate significant differences (*p* < 0.05).

**Figure 3:**
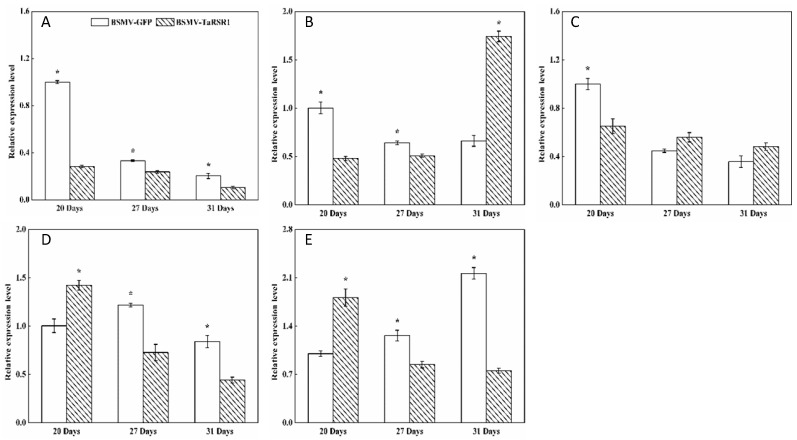
Transcription levels of the AGPase starch synthesis-related enzyme genes in wheat grains of BSMV-TaRSR1-infected and BSMV-GFP-infected spikes at 20, 27 and 31 days after anthesis. Transcription levels were measured by qPCR using *Actin* gene as internal control. (**A**–**E**), transcription levels of *TaAGPS1-a*, *TaAGPS1-b*, *TaAGPS2*, *TaAGPL1* and *TaAGPL2* genes, respectively. Each value is the mean ± standard deviation of three independent biological replicates. Asterisks indicate significant differences (*p* < 0.05).

**Figure 4:**
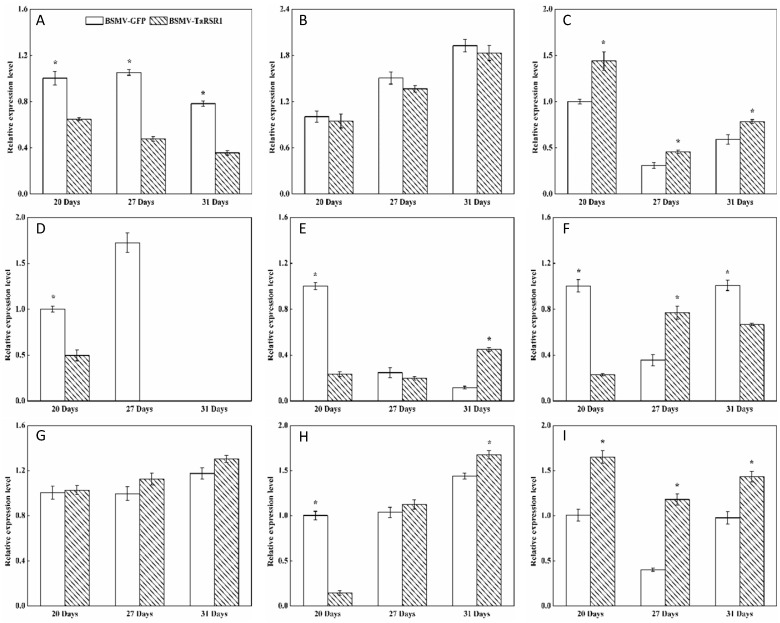
Transcription levels of the GBSS and SS starch synthesis-related enzyme genes in wheat grains of BSMV-TaRSR1-infected and BSMV-GFP-infected spikes at 20, 27 and 31 days after anthesis. Transcription levels were measured by qPCR using *Actin* gene as internal control. (**A**–**I**), transcription levels of *TaGBSSI*, *TaGBSSII*, *TaSSI*, *TaSSIIa*, *TaSSIIb*, *TaSSIIc*, *TaSSIIIa*, *TaSSIIIb* and *TaSSIV* genes, respectively. Each value is the mean ± standard deviation of three independent biological replicates. Asterisks indicate significant differences (*p* < 0.05).

**Figure 5:**
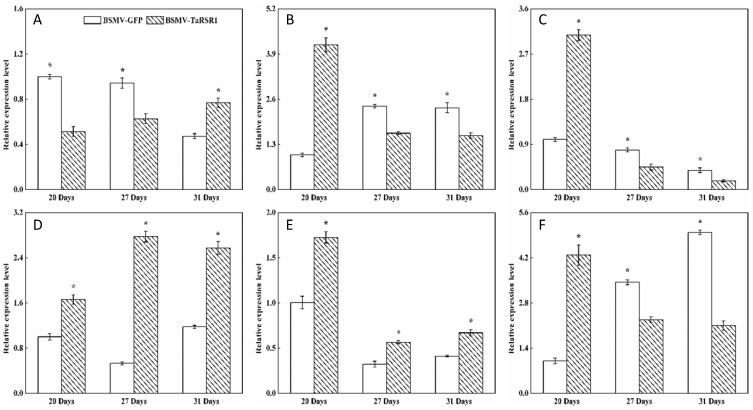

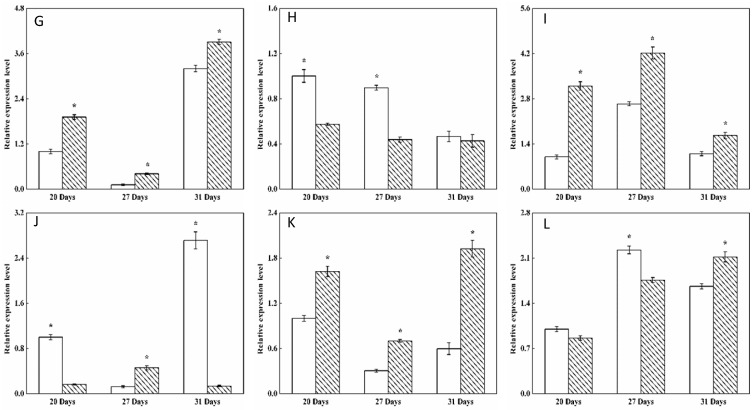
Transcription levels of the branching enzyme (BE), debranching enzyme (DBE), phosphorylase (PHO) and disproportionating enzyme (DPE) starch synthesis-related enzyme genes in wheat grains of BSMV-TaRSR1-infected and BSMV-GFP-infected spikes at 20, 27 and 31 days after anthesis. Transcription levels were measured by qPCR using *Actin* gene as internal control. (**A**–**L**), transcription levels of *TaBEI*, *TaBEIIa*, *TaBEIIb*, *TaBEIII*, *TaISA1*, *TaISA2*, *TaISA3*, *TaPUL*, *TaPHOL*, *TaPHOH*, *TaDPE1* and *TaDPE2* genes, respectively. Each value is the mean ± standard deviation of three independent biological replicates. Asterisks indicate significant differences (*p* < 0.05).

The starch contents, 1000-kernel weights, grain length and width in grains of BSMV-TaRSR1-infected wheat spikes were 30.0%, 19.5%, 14.8% and 8.2% higher, respectively, than those in BSMV-GFP-infected wheat spikes (Figure 2). These results suggest that *TaRSR1* silencing resulted in enhanced starch synthesis in wheat grains; thus *TaRSR1* may act as a negative regulator of starch synthesis, thereby playing a crucial role in the regulation of starch metabolism in bread wheat. The transcription levels of 26 starch synthesis-related enzyme genes in grains of BSMV-TaRSR1 and BSMV-GFP infected wheat spikes at the three sampling time points were also measured by qPCR, with the *Actin* gene used as an internal control (Figure 3, Figure 4 and Figure 5). Similar results were obtained using the *GAPDH* gene as another internal control, as shown in Figure S3.

### 2.4. Transcription Levels of 26 Starch Synthesis-Related Enzyme Genes in BSMV-TaRSR1 Infected Wheat Plants

Some transcription factors can temporally regulate the expression of target genes [36,37]. Our results showed that, in grains of BSMV-TaRSR1-infected wheat spikes, transcription levels of *TaAGPS1-a* (Figure 3A), *TaAGPS2* (Figure 3C), *TaGBSSI* (Figure 4A), *TaSSIIa* (Figure 4D), and *TaPUL* (Figure 5H) genes were strongly downregulated or unchanged at one or more sampling time points. The transcription levels of *TaGBSSII* (Figure 4B) and *TaSSIIIa* (Figure 4G) did not show significant changes; however, *TaSSI* (Figure 4C), *TaSSIV* (Figure 4I), *TaBEIII* (Figure 5D), *TaISA1* (Figure 5E), *TaISA3* (Figure 5G), *TaPHOL* (Figure 5I), and *TaDPE1* (Figure 5K) genes were markedly upregulated at each of the three sampling time points. *TaAGPL1* (Figure 3D), *TaAGPL2* (Figure 3E), *TaBEIIa* (Figure 5B), *TaBEIIb* (Figure 5C), and *TaISA2* (Figure 5F) genes at 20 days; *TaSSIIc* (Figure 4F) and *TaPHOH* (Figure 5J) at 27 days; and *TaAGPS1-b* (Figure 3B), *TaSSIIb* (Figure 4E), *TaSSIIIb* (Figure 4H), *TaBEI* (Figure 5A), and *TaDPE2* (Figure 5L) genes at 31 days were markedly induced, but their transcription levels were repressed or unchanged at the other two sampling time points. These results suggest that *TaRSR1* negatively and temporally regulates the expression of several starch synthesis-related enzyme genes (*TaAGPS1-b*, *TaAGPL1*, *TaAGPL2*, *TaSSI*, *TaSSIIb*, *TaSSIIc*, *TaSSIIIb*, *TaSSIV*, *TaBEI*, *TaBEIIa*, *TaBEIIb*, *TaBEIII*, *TaISA1*, *TaISA2*, *TaISA3*, *TaPHOL*, *TaPHOH*, *TaDPE1*, and *TaDPE2*) and plays a role in wheat starch synthesis. The decreased transcription levels of these starch synthesis-related enzyme genes at one or more sampling time points may be due to the increased transcription of their isoform genes, or these genes may be positively regulated by TaRSR1; however, further studies are required. Similar expression patterns of starch synthesis-related enzyme genes have been reported in rice overexpressing the OsbZIP58 transcription factor [38].

The number (19) of regulated starch synthesis-related enzyme genes in the grains of BSMV-TaRSR1-infected wheat spikes was determined (Figure 3, Figure 4 and Figure 5), and found to be greater than that (11) of starch synthesis-related enzyme genes whose transcription levels showed a negative relationship with TaRSR1 [7]. In addition, only seven starch synthesis-related enzyme genes (*TaAGPL1*, *TaSSI*, *TaSSIV*, *TaBEI*, *TaBEIIb*, *TaPHOL*, and *TaDPE1*) had similar expression profiles in both experiments. The differences between these two studies may mainly be because the BSMV-VIGS method used in this study more accurately determined the function of target genes than the correlation analysis used in our previous study [7]. In addition, differences may be partially due to the different sampling time points between this study (21, 27, and 31 days after anthesis) and previous studies (5, 10, 15, 20, 25, 30, and 35 days after anthesis).

### 2.5. Putative Mechanism of TaRSR1 Regulation in Higher Plants

In higher plants, APETALA2/ethylene-responsive element binding protein (AP2/EREBP) is one of the largest families of transcription factors [39]. The AP2/EREBP superfamily is divided into AP2, ethylene-responsive transcription factor, and related ABI3/VP1 families [40,41]. The *RSR1* transcription factor belongs to the AP2 group of the AP2/EREBP family, which is characterized by an AP2 domain [20]. AP2/EREBP can recognize the GCAC(A/G)N(A/T)TCCC(A/G)ANG(C/T), DRE, GCC, JERE, CAACA, ACGT, and ACTCAT boxes in the promoters of target genes [38,42,43,44,45,46]. These boxes have been identified in the promoters of some upregulated starch synthesis-related enzyme genes in grains of BSMV-TaRSR1-infected wheat spikes. For example, ACGT and ACTCAT boxes were found in the promoters of *GBSSI* and *BEI* genes of rice [38,45], and *AGPS1*, *SSI*, *SSIIIa*, and *ISA1* genes of maize, respectively [46]. Therefore, *TaRSR1* may directly and negatively regulate the expression of some starch synthesis-related enzyme genes.

## 3. Materials and Methods

### 3.1. Plant Materials

Bread wheat cultivar (Bainong 207, a high-yield wheat cultivar developed by Henan University of Science and Technology, Xinxiang, China) was grown in the Agricultural and Experimental Farm of Henan Agricultural University (Zhengzhou, China, N34°9′, E113°6′) during the wheat growing season (October 2014 to May 2015). Type of soil, organic matter, nitrogen, phosphorus, and potassium, fertilizer application, and planting management were reported in our previous study [8]. Seeds were sown on 16 October 2014, and the plant density was adjusted to 150 plants/m^2^ at the three-leaf stage. The plot dimension was 4 m × 8 m, and plots were separated by a ridge (20 cm in width). Wheat plants with a 15 m^2^ area (length 5.0 m × width 3.0 m) were covered with an arch plastic shed (3.0 m width × 1.5 m height × 0.5 m arch height) during the heading stage, and light transmittance of the polyvinylchlorid (PVC) film (Auspicious dragon import and export trade company, Zhejiang, China) was 75%. Ninety wheat spikes with the same anthesis date (18 April 2015) in the arch plastic shed, which was built with concrete pillars and bamboo poles, were marked. At 1 day before inoculation, the surrounding PVC plastic film was covered with soil to prevent water loss and increase humidity in the shed. Developing grains in the middle of BSMV-infected spikes were sampled at 20, 27, and 31 days after anthesis at 11:00 in the morning. The sampled grains were rapidly frozen in liquid nitrogen for 2 min and then stored at −80 °C for transcription analysis of starch synthesis-related enzyme genes. During the mature stage, grains of BSMV-infected wheat plants were harvested and dried to a constant weight at 70 °C in the oven for starch determination.

### 3.2. Construction of BSMV-Derived Vectors

Because of asymmetric expression patterns of two or more homoeoalleles in allopolyploid plants [17], only one copy (Genome location: 1DS) of *TaRSR1* could be expressed in grains of bread wheat in our previous study [7]. In this study, the full-length cDNA sequence of this copy, its conserved cDNA fragment (284 bp) used to construct the BSMV-TaRSR1 vector, and the other two copies (1AS and 1BS) in bread wheat A and B subgenomes are indicated in Figure S1. Because there are high similarities in cDNA or protein sequences among three or more copies of a gene in allohexaploid bread wheat, VIGS method usually results in the simultaneous silencing of three copies for the targeted gene in bread wheat [47]. In this study, similarly, the chosen cDNA fragment of TaRSR1 (1DS) for VIGS silencing also had high similarities (99.1%) to those of the other two copies (Figure S1), and thus, all three copies of *TaRSR1* gene could be simultaneously silenced in this study. The forward and reverse primers were 5′-CTAGCTAGCGCCTGCAACTCCACCATG-3′ and 5′-CTAGCTAGCCGGACGATGACGACGAGA-3′, respectively. The amplified cDNA sequence of the *TaRSR1* gene fragment was sequenced by Applied Biosystems and the correct TaRSR1 sequence was cloned into the BSMV plasmid using an *Nhe* I site. The schematic construction of recombinant BSMV-TaRSR1 vector is described in Figure S4. Similar to a study by Pacak et al. [48], *GFP* was used as a control in this study.

### 3.3. In Vitro Transcription of Viral RNAs and Plant Inoculation

The in vitro transcription of viral RNAs was performed as described by Ma et al. [49]. The RNA-α, RNA-β, and RNA-γ-derivative clones were linearized with *Mlu* I and *Spe* I, respectively. RNA synthesis was performed using the RiboMAX™ Large Scale RNA Production System-T_7_ kit (Promega, Fitchburg, WI, USA) according to the manufacturer’s instructions. The RNA-α, RNA-β, and RNA-γ (or its derivative) transcripts were mixed in a 1:1:1 ratio, and subsequently diluted with nine volumes of DEPC water. In addition, 12 volumes of 2× GKP buffer were added to the diluted transcript mixture for subsequent inoculation. To increase humidity, wheat plants in the shed were sprayed with distilled water until it ran off at 1 day before inoculation. Virus inoculation was performed on the spike of wheat plants during the heading stage and was accomplished by gently rubbing the spike surface five times from the central section of wheat spikes to the tip and then from base to tip. Wheat spikes were inoculated with BSMV-TaRSR1 or BSMV-GFP transcript mixtures with a gloved finger (Figure S5). A total of 45 plants were inoculated with BSMV-TaRSR1 or BSMV-GFP, respectively, and 20 µL BSMV-TaRSR1 or BSMV-GFP transcript mixtures were used for each spike. After inoculation, BSMV-inoculated spikes were fog-sprayed with nuclease-free water and covered with plastic film to maintain high relative humidity (85.0%) for 24 h, and wheat plants remained covered with the arch plastic shed for 7 days and two sides were opened during the daytime due to high temperatures and closed at night to maintain high humidity.

The manufacturer’s instructions were followed and treated (Takara Biotechnology (Dalian) Co., Ltd., Dalian, China) to remove contaminating. First-strand cDNAs were synthesized from 2 µg of total RNA using Super-Script II reverse transcriptase (Invitrogen, Carlsbad, CA, USA).

### 3.4. Determination of Transcription Levels of TaRSR1 and 26 Starch Synthesis-Related Enzyme Genes

Total RNA was extracted from the grains of BSMV-TaRSR1-infected or BSMV-GFP-infected wheat spikes with TRIzol reagent (Invitrogen, Carlsbad, CA, USA) and genomic DNA was removed with RNase-free DNase I. The first strand cDNA was synthesized from 2 µg of total RNA using the Revert Aid™ First Strand cDNA Synthesis Kit (Fermentas, Burlington, ON, Canada). Transcription levels of *TaRSR1* and 26 starch synthesis-related enzyme genes were measured using the qPCR method, which was performed on a Light Cycler 480 Real-Time PCR System (Roche Diagnostics Ltd., West Sussex, UK) using an SYBR Premix Ex Taq (Perfect Real Time) Kit (Takara Biotechnology Co., Ltd., Dalian, China). Each reaction consisted of 10 µL SYBR Green Supermix (2×), 1 µL diluted cDNA, 0.5 µL forward primer, and 0.5 µL reserve primer, in a total volume of 20 µL. The relative transcription levels of each starch synthesis-related enzyme gene were calculated using the 2^−^^ΔΔ*C*t^ method, with wheat glyceraldehyde 3-phosphate dehydrogenase (GAPDH) (GenBank accession no. EF592180) and *β-actin* (GenBank accession no. AB181991) genes as the internal controls. All of primers used for qPCR are indicated in Table S1.

### 3.5. Assays on Wheat Grains Parameters

Starch contents were measured according to the methods of Zhao et al. [50]. Grain length and width were determined by calculating ten grains from each plant. The 1000-kernel weight was determined by counting four replicates of 100-grain samples independently on an electronic balance. Fifty seeds were analyzed for seed size, and data are presented as means ± SD.

### 3.6. Statistical Analysis

Data were statistically analyzed using Student’s *t*-test with SPSS Statistics 17.0 (SPSS Inc., Chicago, IL, USA) and significant differences among group means was tested by using Duncan’s multiple range test at *p* < 0.05 level. The averaged values of three spikes were considered one replicate and three independent biological replicates were performed. All of the recorded values represent the means of the results from three independent biological replicates.

## 4. Conclusions

In this study, the *TaRSR1* transcription factor was silenced in grains of wheat plants using the BSMV-VIGS method under field conditions. In BSMV-TaRSR1-infected wheat plants, starch contents, 1000-kernel weights, grain length and width markedly increased in mature grains. In addition, the transcription levels of 19 starch synthesis-related enzyme genes were significantly upregulated at one or more sampling time points in the grains of the infected wheat plants. These results indicate that TaRSR1 acts as a negative regulator and temporally regulates the expression of some starch synthesis-related enzyme genes in bread wheat.

## Supplementary Materials

Supplementary materials can be found at www.mdpi.com/1422-0067/17/9/1557/s1.
