# Supplementary material for: Virus-Induced Gene Silencing Identifies an Important Role of the TaRSR1 Transcription Factor in Starch Synthesis in Bread Wheat
Source: Int J Mol Sci. 2016 Sep 23;17(10):1557. doi: 10.3390/ijms17101557 (PMC5085620; doi:10.3390/ijms17101557)
Supplement: Supplementary file 1 [file ijms-17-01557-s001.pdf]

Supplementary Materials: Virus-Induced Gene Silencing Identifies an Important Role of the TaRSR1 Transcription Factor in Starch Synthesis in Bread Wheat

Guoyu Liu, Yufang Wu, Mengjun Xu, Tian Gao, Pengfei Wang, Lina Wang, Tiancai Guo and Guozhang Kang

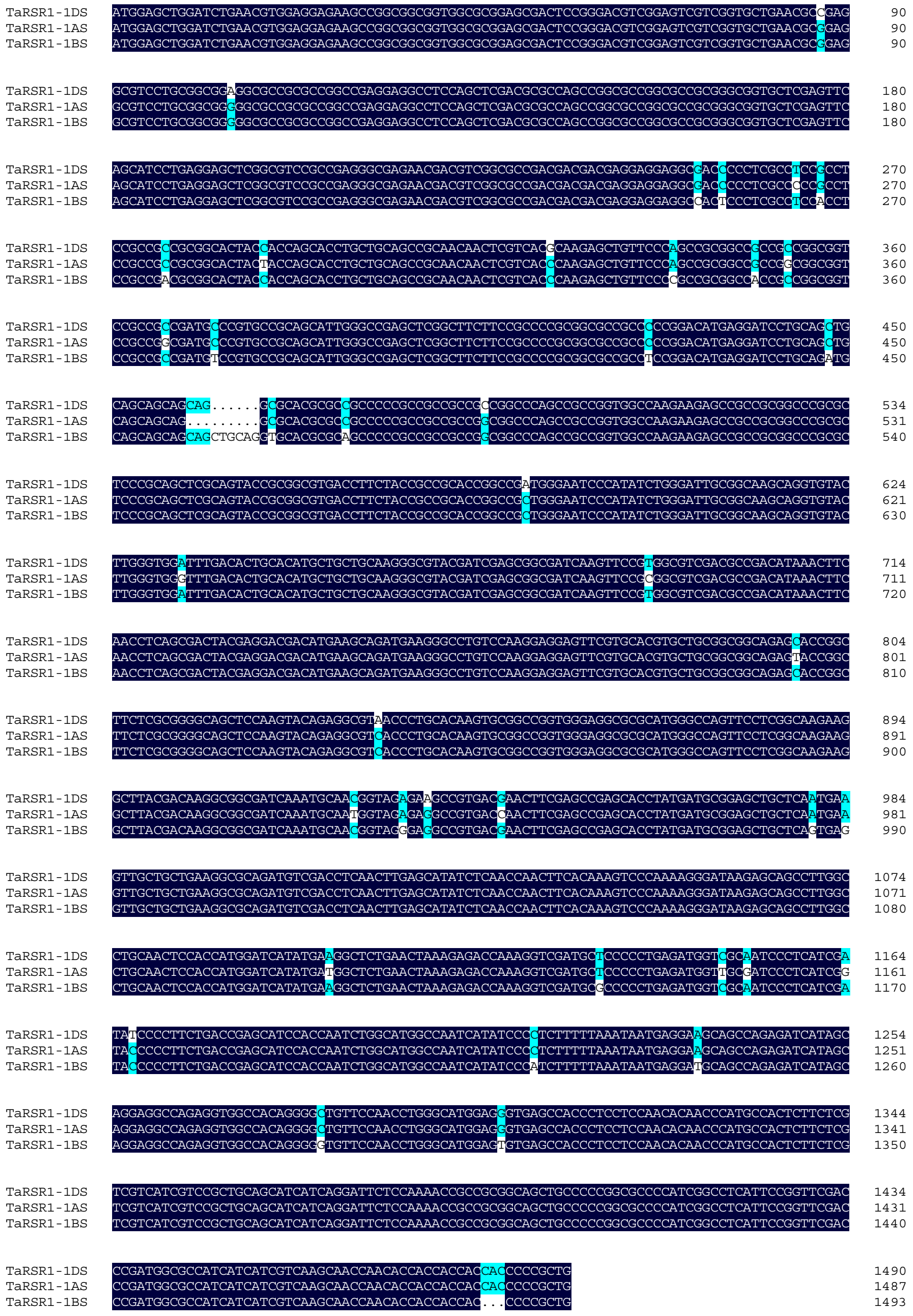

Figure S1. cDNA sequences for three copies of *TaRSR1* gene. The fragment used to VIGS silencing has been underlined. Base pairs signed with black or blue colors are defined as same bases between two or three copies. White pairs signed with white color are defined as different bases between one copy and the other two copies.

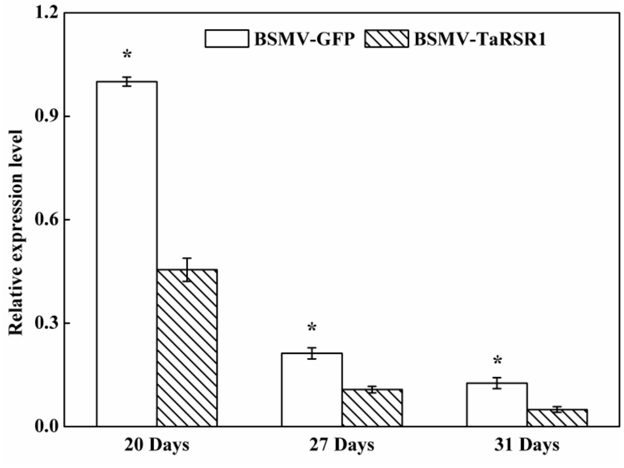

Figure S2. Transcription levels of the *TaRSR1* gene in the BSMV-TaRSR1-infected and BSMV-GFP-infected wheat plants at 20, 27 and 31 days after anthesis. Transcription levels were measured by qPCR using *GAPDH* gene as internal control. Each value is the mean ± standard deviation of three independent biological replicates. Asterisks indicate significant differences ($p < 0.05$).

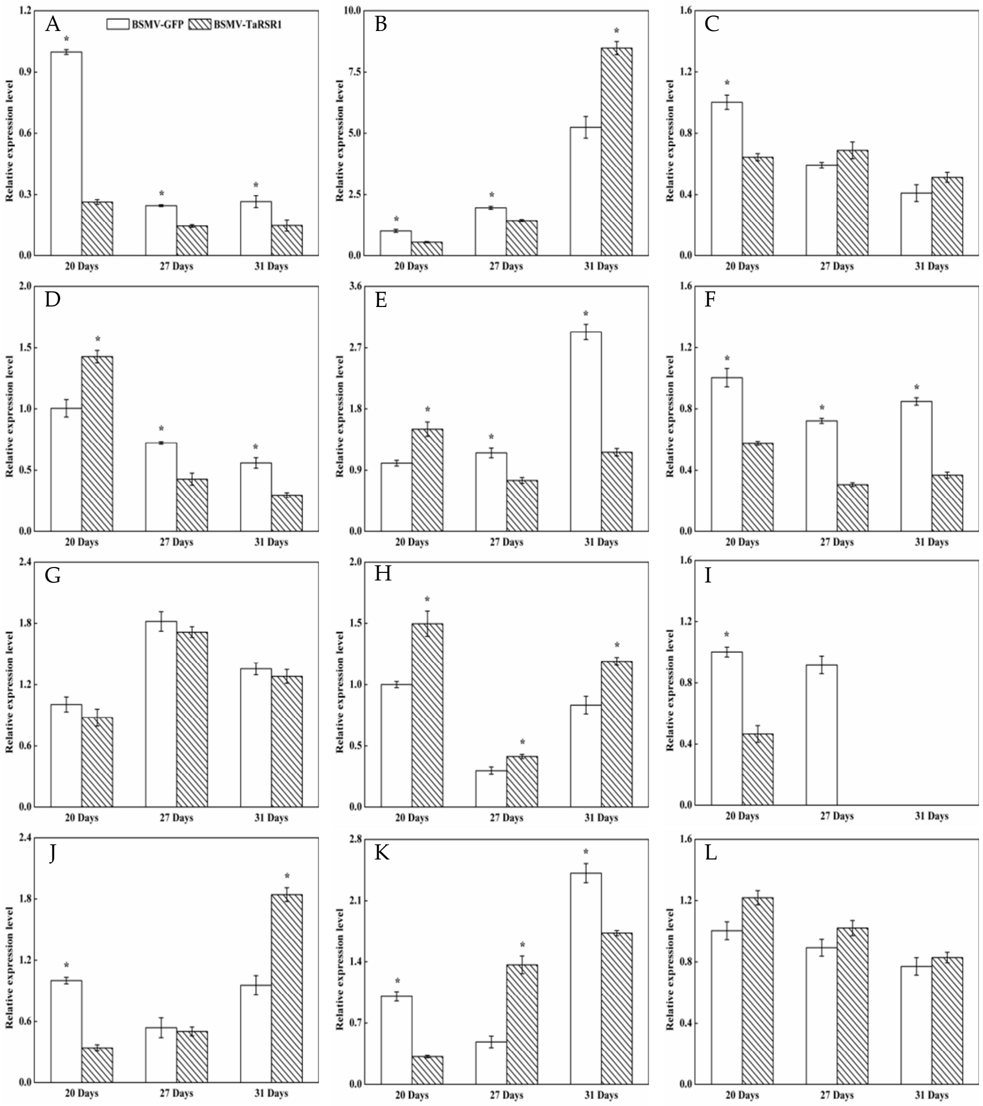

Figure S3. *Cont.*

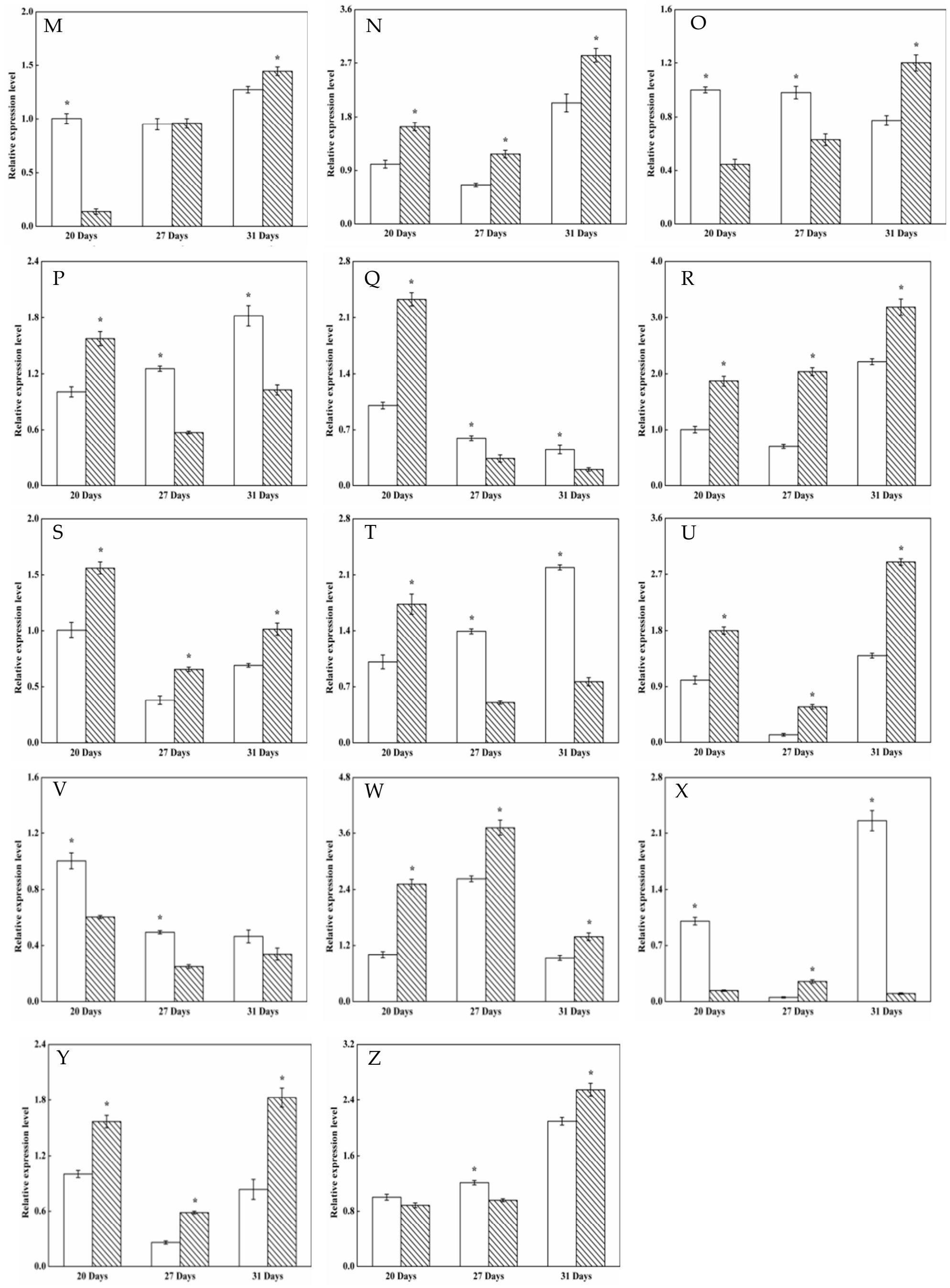

Figure S3. Transcription levels of the 26 starch synthesis-related enzyme genes in the grains of BSMV-TaRSR1-infected and BSMV-GFP-infected plants at 20, 27 and 31 days after anthesis. Transcription levels were measured by qPCR using *GAPDH* gene as internal control. (**A**–**Z**), transcription levels of *TaAGPS1-a*, *TaAGPS1-b*, *TaAGPS2*, *TaAGPL1*, *TaAGPL2*, *TaGBSSI*, *TaGBSSII*, *TaSSI*, *TaSSIIa*, *TaSSIIb*, *TaSSIIc*, *TaSSIIIa*, *TaSSIIIb*, *TaSSIV*, *TaBEI*, *TaBEIIa*, *TaBEIIb*, *TaBEIII*, *TaISA1*, *TaISA2*, *TaISA3*, *TaPUL*, *TaPHOL*, *TaPHOH*, *TaDPE1*, and *TaDPE2* genes, respectively. Each value is the mean ± standard deviation of three independent biological replicates. Asterisks indicate significant differences ($p < 0.05$).

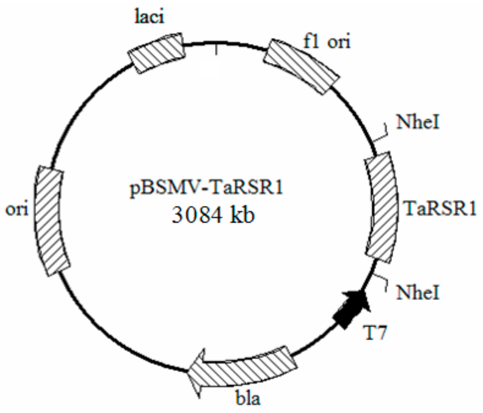

Figure S4. Schematic construction of the recombinant barley stripe mosaic virus-wheat starch regulator 1 (BSMV-TaRSR1) vector.

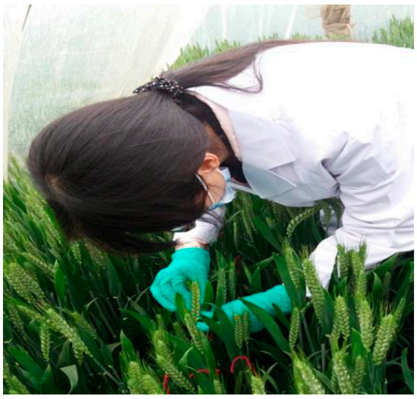

Figure S5. Barley stripe mosaic virus-wheat starch regulator 1 (BSMV-TaRSR1) and barley stripe mosaic virus-green fluorescent protein (BSMV-GFP) virus inoculation under field conditions.

Table S1. Target genes for analyzing transcription levels and their primer sequences.

Gene Name	Accession Number (Acc. No.)	Forward Primer (5′–3′)	Reverse Primer (5′–3′)	Amplification Sizes Base Pair (bp)
TaAGPS1-a	X66080	CCTTCCAAGCGTGAACAA	TTCCGAGAACACTATCATCAAC	94
TaAGPS1-b	EU586278	AAGATCCTGATCCCTCCG	CTAGTCCCTGCACCACCT	140
TaAGPS2	AY727927	TAATTCCGAGCGGGACAG	AAATGGTGCCTTGAGTGG	199
TaAGPL1	Z21969	GCCCCTGTTGGAGAGAGCCG	TAGCAGGGTCGTCGATGGCG	157
TaAGPL2	DQ406820	ATTGATGGCAGCCGGGCGTC	TCGGTAAGCCGAGGAGAGTGGT	136
TaGBSSI	AF286320	CGGCATGGACGTCAGCGAGT	AGGGGCACCTTCCGGTCCAC	147
TaGBSSII	AF109395	TGCATTGGAGGCTCCGAGGGT	GCAGTGTGCCAGTCATTTGCAACG	102
TaSSI	AJ292521	GAACTGGGGGCCTCCGAGACA	CCTCCCAGGACGGCTTGTGC	162
TaSSIIa	AJ269503	CACGCCGGTGAACGGTGAGA	CGGCGAGGCGACGTTAGCTT	148
TaSSIIb	EU333947	GCCAGAAGAGGGCATCGCGT	ATTGTGCCGGTGGCGGAAGG	189
TaSSIIc	EU307274	GGTCTCCCGGTTCGTGGGGA	TTGCCTCCCGGTACCCAGCA	144
TaSSIIIa	AF258608	CGGTTGACGAAGTCGGGCCT	CCCAGCTTCATCCACTGCACCC	197
TaSSIIIb	EU333946	GCAGCGTGGTGCTGGAGGTAG	TGATTGGTCCTGTCCTTGGGTGC	176
TaSSIV	AY044844	CGACGAGCAGGGCCTAAGCA	AGCCGAGGTGTCCCAGCTGAA	124
TaBEI	Y12320	TGGGTCGATCGGGTTCCTGCAT	ACGTGGAGCGTCAGGCTTTCG	150
TaBEIIa	AF286319	GCAAGTCCGGCGCAACCTGA	CTCGCGGTTTCTCCCCCACG	190
TaBEIIb	AY740401	CGCCTTCCATCGACGGTCCC	TCCCGGTGGTGGCAGAATGC	212
TaBEIII	JQ346193	TACGTTGACAAGGATGCGCT	CTAATCCGCCTTGGGTGGTT	139
TaISA1	AF548380	AGTACCGGGACATTGTGCGCC	GCCAAGGTTTCCTTCCTCCTGCC	117
TaISA2	JX473824	GCCACGTCTGGCACGTCTCG	TGGCAGCAACCAGGTCACCG	132
TaISA3	JN412069	CCTTGGGATTGTGGAGGTCTTT	CATGATGAGGCTTCCGCTGGTT	193
TaPUL	EF137375	CCCTCCTCAGTCCCAGGTGT	TGCGGACACACATTGAAGACCG	123
TaPHOL	EU595762	TGGGGATCATGCCAAGGCCAA	CCTGCAGGGAGGCCGAGCAT	140
TaPHOH	AF275551	GGCCAGCAAAAAGCGCCTAGC	TGCCTGTCTGCTGCGCTCAT	180
TaDPE1	DQ068045	GGATGTCTCTTGGGCGCTAA	TGCTTCTTCGAGGCTAAGGC	187
TaDPE2	BQ294920	AGTATTCGGCGTGGTCTGTTCG	TGCTATGTCATCCAAGTCCCTA	132
TaRSR1	JX473823	GCCTGCAACTCCACCATG	CGGACGATGACGACGA	284
TaActin	AB181991	AAACGAAGGATAGCATGAGGAAGC	AGCGGTCGAACAACTGGTA	101
TaGAPDH	EF592180	TTTTCACCGACAAGGACA	AAGAGGAGCAAGGCAGTT	179
